# Affective Recommender System for Pet Social Network

**DOI:** 10.3390/s22186759

**Published:** 2022-09-07

**Authors:** Wai Khuen Cheng, Wai Chun Leong, Joi San Tan, Zeng-Wei Hong, Yen-Lin Chen

**Affiliations:** 1 Faculty of Information and Communication Technology, Universiti Tunku Abdul Rahman, Kampar 31900, Perak, Malaysia; 2 Department of Information Engineering and Computer Science, Feng Chia University, Taichung 40724, Taiwan; 3 Department of Computer Science and Information Engineering, National Taipei University of Technology, Taipei 106344, Taiwan

**Keywords:** affective recommendation, pet social network, emotion recognition model, dog barking recognition, deep learning

## Abstract

In this new era, it is no longer impossible to create a smart home environment around the household. Moreover, users are not limited to humans but also include pets such as dogs. Dogs need long-term close companionship with their owners; however, owners may occasionally need to be away from home for extended periods of time and can only monitor their dogs’ behaviors through home security cameras. Some dogs are sensitive and may develop separation anxiety, which can lead to disruptive behavior. Therefore, a novel smart home solution with an affective recommendation module is proposed by developing: (1) an application to predict the behavior of dogs and, (2) a communication platform using smartphones to connect with dog friends from different households. To predict the dogs’ behaviors, the dog emotion recognition and dog barking recognition methods are performed. The ResNet model and the sequential model are implemented to recognize dog emotions and dog barks. The weighted average is proposed to combine the prediction value of dog emotion and dog bark to improve the prediction output. Subsequently, the prediction output is forwarded to a recommendation module to respond to the dogs’ conditions. On the other hand, the Real-Time Messaging Protocol (RTMP) server is implemented as a platform to contact a dog’s friends on a list to interact with each other. Various tests were carried out and the proposed weighted average led to an improvement in the prediction accuracy. Additionally, the proposed communication platform using basic smartphones has successfully established the connection between dog friends.

## 1. Introduction

With the emergence of the Internet of Things (IoT), the landing of smart homes in the new era is no longer impossible. Current smart home designs are smarter when integrated with recommender systems (RS) [1,2,3,4,5,6,7,8]. RS and the Internet of Things (RSIoT) are highly dependent on real-time resources, especially sensor data, not just interactions between users and items. The initial stages of acquiring data, especially from sensors, are critical as these data are preprocessed (removing noise or redundant features) and generated events by defining suitable rules. After that, the system is able to learn the pattern of the rules and provide recommendations that match users’ preferences. Some smart systems [9,10,11,12,13] have been developed to promote efficient resource mapping through user habits. Habits are often formed when intentions are translated into actions and behaviors repeatedly [14]. Resource mapping efficiency can be achieved by gradually changing user habits through micro-moments and recommendations [15]. Most current systems are smarter than ones in the past because they leverage users’ social networks and integrate this information with the system to provide preferred recommendations [11]. Furthermore, by considering the characteristics of users, a preferred system with an appropriate level of automation can be designed [16].

Users of smart homes are not limited to humans but animals. Most pet owners can only monitor their pets’ behaviors through home security cameras, especially when they are not at home. However, some pets such as dogs require long-term close companionship with their owners. Dogs are highly social animals that easily form close attachments to their own species or other species [17]. There comes a time when a puppy or dog is separated from its owners and most of them learn to adjust to social isolation at home. However, some dogs later become sensitive to social isolation (left at home alone) and tend to develop separation anxiety, which can lead to excessive vocalization and disruptive behavior. Huasang et al. [18] proposed a multi-level hierarchical behavior monitoring system to detect separation anxiety symptoms in dogs. The purpose of the system is to automatically monitor the dogs and analyze their behaviors through a taxonomy consisting of three progressive levels. In the system, the Stacked Long Short-Term Memory (LSTM) is adopted to recognize postures through sensors. These postures are then interpreted by a Fuzzy Complex Event Processing (CEP) engine that detects the anxiety symptoms.

In this study, we developed a smart environment for domestic dogs that not only monitors dogs’ behaviors but also integrates their social networks to relieve separation anxiety, especially for those that are left alone. People adopted dogs for stress reliever, companion, and protection purposes [19]. This phenomenon is more pronounced during the COVID-19 pandemic period. However, pandemic puppies turn into a big issue for many inexperienced owners. These puppies are deprived of socialization, which not only happens during the pandemic but gets worse once their inexperienced owners return to normal job routines as before the pandemic. After the lockdowns are lifted, dogs need a transition period to get used to being away from their owners. According to suggestions by dog behavior specialists and veterinarians [20,21,22], there are several ways to ease post-pandemic separation anxiety in dogs. Experts recommend dog owners to provide an environment in which the dogs can relax when nobody is home. Dog owners can also adopt some technology gadgets to monitor their dogs and make those gadgets as interactive toys to keep their brains and bodies moving when they are alone. All these approaches are useful in providing dogs with enrichment that can be enjoyed independently. Our proposed system aims to create a safe and comfortable place for dogs and implement the suggested approaches by the experts in solving dogs’ separation anxiety issues. The proposed solution allows dogs from different households to communicate remotely by using a distributed system architecture with cloud computing adoption. The Real-Time Messaging Protocol (RTMP) servers are used by the social network platform to connect and communicate with their dog friends. In the system, on the other hand, the dogs’ behaviors are predicted through emotion recognition and sound (barking) recognition, and this makes it possible to implement an efficient recommender system for dogs. A large number of images consisting of various dog expressions were collected and a dog expression classification model was trained using the Residual Neural Network (ResNet) [23]. Dogs’ emotions are definable based on predicted expressions. Similarly, different audio files of dog barks were collected, and the sequential model was used to train a sound recognition classification model. Subsequently, the expression classifier was combined with the sound classifier using weighted average techniques to improve the behavior prediction results.

The main contributions of this paper are summarized as follows: (1) present a unique cloud-based smart environment dogs’ social network architecture; (2) propose an affective recommender framework with dogs’ emotion recognition and sound (barking) recognition; (3) proof of concept and verify the viability of the proposed dogs’ social network architecture. The rest of the paper is organized as follows. In Section 2, related works on the RSIoT are presented. Section 3 illustrates the overall cloud-based dogs’ social network architecture. The affective recommender framework and the dogs’ emotion recognition model are discussed in Section 4. Section 5 presents the experiment results, and the conclusion is stated in the last section.

## 2. Related Work

With the advancement of technology and the pursuit of a better quality of life, smart home systems are rapidly gaining attention. The main purpose of most systems is to identify any proactive behavior of users in the current situation and recommend them a service that suits their habits [1]. The recommendations are constructed based on long-term studies of the repetitive patterns in users’ daily lives [3]. In 2010, Parisa et al. [24] developed an unsupervised model to track and recognize activities in a smart environment. The Discontinuous Varied-Order Sequential Miner (DVSM) was proposed to determine activity patterns that might be discontinuous or in various order. The patterns were grouped together and represented using cluster centroids. Later, the boosted version of the hidden Markov model was used to represent the activities and recognize them in the environment.

Katharina [1] proposed a smart home system integrated with an unsupervised recommender system that predicted the relationships between users’ actions through collected data. The system tries to predict the next action of the users and recommends some actions. Firstly, a formal model of the context which represents the multidimensional space was constructed. These contexts were the users’ actions that related to each other which integrated with time elapsed and represented with tuples. These tuples were trained based on the basis of observed sensor events. An algorithm Dempster–Shafer theory which is similar to the Naïve Bayes was proposed to predict the next contexts based on the current action. A ranked list was provided as the output of the recommendation.

The Pervasive RS (PRS) was proposed by Naouar et al. [25] which represents the contexts in tuples. The data were collected through physical sensors including RFID, and later it was transformed into various contexts to build the user profile according to preferences. Preferences are actions that occur repeatedly and are relevant to each other. The Apriori algorithm was implemented to extract the relevant preferences that occurred from the database. A three-layer neural network based on back propagation was proposed to predict user preferences in a given context. Nirmalya and Chia proposed a model named Complex Activity Recognition Algorithm (CARALGO) which is based on probability theory [26]. The main idea is to decompose a complex activity into small atomic activities, and the context attributes are constructed so that each of these activities is associated with a specific weight depending on their relevance. The occurrence of the activities is decided by the threshold function. The number of ways to perform complex activities is derived through the binomial theorem.

Alexander et al. [27] discussed the new recommendation techniques that are relevant to real-world IoT scenarios including the IoT gateway. Smart homes with RS should be able to enhance the applicability of the equipment and optimize the usage of the resources. The SEQREQ was developed to recommend items by finding sequential patterns; it analyzes users with similar behaviors that share common sequences of actions. The idea is to find the common node sequences (which are similar to the actions) that are available in the workflow repository and list them in a look-up table. Then, similarity values are calculated between the actions and the common node sequences where values greater than zero will be recommended. It is important that the RS is able to recommend items based on the sequence of the activities.

The subjects of recognition are not limited to humans; they can also be animals such as horses [28]. The behaviors of both subjects were analyzed in order to recognize their actions and provide some recommendations. In terms of Animal Activity Recognition (AAR), it can be an owner that is monitoring their pet when they are not at home; it can also be the observation of wildlife in a natural environment. Basically, the processing pipeline of the AAR and the Human Activity Recognition (HAR) are quite similar to each other since they both capture the activity data through sensors, and the features from the activity data are extracted and further classified into a few groups [29]. The main difference between the AAR and the HAR is the input data and the output data they produce.

Cassim et al. [30] carried out a study to recognize the activity of dogs. It determines a set of activities that are connected to the behavioral patterns that identify dogs’ behavior. The dogs were required to wear a collar-worn accelerometer in order to collect their movements, such as body movements and response behaviors. Feature extraction was carried out using principal component analysis (PCA) and the k-nearest neighbor was implemented to classify the features. Yumi et al. [31] proposed research to study the AAR based on a first-person view from a dog. In this research, a GoPro camera was attached to the back of the dogs and recorded the activities that were carried out by them from their viewpoint. From the video recording, global and local features were extracted using various algorithms such as dense optical flow, local binary patterns, cuboid detector, and STIP detector. Global features were mainly captured from the dogs’ motions, whereas local features were captured from motions other than the dogs. Visual words were integrated in order to increase the efficiency of the representation of the motion. Lastly, the support vector machine (SVM) is used to classify first-person animal activities through features.

Patricia, Javier, and Alejandro [32] developed a system that is able to track cats’ location, posture, and field of view using a depth-based method. The Microsoft Kinect sensor, which is able to record both color and depth video, was set up to capture the motion of a cat. The depth value of a cat’s pixel in each video frame was extracted and divided into different clusters using the k-mean algorithm. Different postures produced different depth values for every part (head, body, and tail) of the cat. A decision tree was constructed by considering different parameters to determine body postures and classify the clusters. Jacob et al. proposed a multitask learning (MTL) framework for embedded platforms to perform AAR [33]. This framework is able to solve multiple tasks simultaneously and explore connections among the tasks using the Relief algorithm. The dataset was collected from multiple sensors and features were extracted. To perform action (or task) classification, seven classification techniques including deep neural network (DNN) were implemented. DNN was able to provide promising results in this approach. Enrico et al. [34] studied horse gait activity recognition by capturing the data using the built-in accelerometer sensor in a smartwatch through a developed application. The smartwatch was placed on the saddle of a horse and the wrist of the rider. Each gait has distinctive characteristics, and its features were extracted using different algorithms such as neural networks, decision trees, k-neighbors, and support vector machines. The performances of the algorithms were compared and showed similar results.

Studies in pet emotion recognition and RS are still under exploration, and most of the existing works are mainly focused on dogs [35] and cats [36]. For instance, Quaranta et al. [36] noticed that different cats’ vocalizations that they had recorded produced different patterns of sound waves. Each pattern of sound waves should represent a relevant cat condition. Similar research was presented by Varun et al. [37]. They presented a recommender framework with dog vocalization pattern recognition in their study. The authors gathered a number of vocalization patterns and taught the convolutional neural networks to recognize dog emotions. Bhupesh et al. [38] noticed that animals express different types of expressions on their faces in different scenarios. The authors managed to run several experiments to assess their hypothesis on sheep and rats. They observed the animals’ noses, ears, whiskers, and eyes react differently when receiving different levels of stimulation. In addition, Cátia Caeiro et al. [39] also inspected dogs’ facial expressions under different scenarios. They discovered dogs showed a higher level of facial expression in conditions such as “fear” and “happy”, but not “frustrated”.

In recent years, deep learning has been widely used for various recognition applications as it is able to provide promising outputs with sufficient training through large amounts of data [40,41,42]. Through the training process, it is able to capture the relationship between the data itself [43]. Mohammed et al. [44] proposed a novel approach that was implemented through the deep belief network (DBN) to train the activities and recognition. The actions were collected using accelerometers and gyroscope sensors. Based on the sensor data, multiple features were extracted, and the kernel principal component analysis (KPCA) was used to reduce the data dimension before training. Jacob et al. [45] studied the AAR by focusing on unsupervised representation learning. It aimed to recognize activities from the raw motion data (unlabeled) that was collected online using an accelerometer. Various features were extracted from the collected data using algorithms and further classified into different activities. Algorithms such as PCA, sparse autoencoders (SAE), and convolutional deep belief network (CDBN) were implemented to extract features, while the support vector machine (SVM) was used to perform the activity classification. The performances of these algorithms were compared and evaluated using F1 measures. Rosalie Voorend [29] implemented a variational autoencoder (VAE) to perform feature extraction and a sequential classifier to classify the activity. The autoencoder was proposed to deal with unsupervised representation learning and it has not been extensively explored in the AAR. However, the output that the autoencoder produced is not satisfying enough when compared to the statistical approach. This is probably because the loss function in the VAE is not optimized. Coherence within the input data which causes the representations to be unable to be extracted properly is needed as well. Enkeleda et al. [46] proposed deep convolutional neural networks (ConvNets) to recognize the activity of livestock animals without feature extraction. The proposed network has four layers and each layer consists of different operations. Different hyperparameters were adjusted and their performances were compared.

## 3. Dogs’ Social Network Architecture

**Figure 1:**
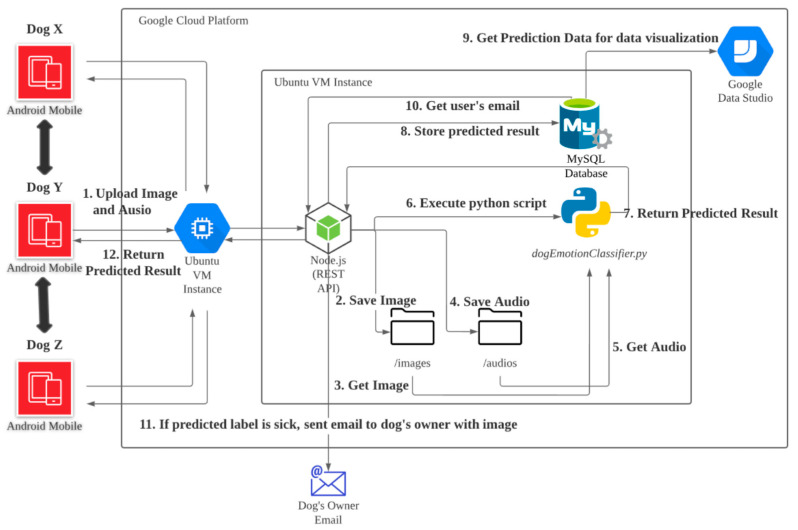
The proposed cloud-based smart environment dogs’ social network architecture.

Figure 1 illustrates the overall implementation of a pet social network on a cloud computing platform. First, an Android app was developed with social networks and sensing capabilities (e.g., cameras and microphones). The social networking app serves as the interface layer to allow owners to register their dogs and connect other users’ profiles to their pets’ networks. The mobile app can detect dogs’ movements and capture their images and sounds via live streaming which is connected to its own Real-Time Messaging Protocol (RTMP) server when the dog is near the device. The captured frames (images and audio clips) will then be uploaded to the Ubuntu VM instance hosted in the Google Cloud Platform. Those images and audio clips are uploaded through POST requests to the Node.js RESTful API. After receiving the files, Node.js saves the image and audio files into the “/images” and “/audios” directories, respectively. The affective recommender engine will be triggered by a python script (dogEmotionClassifier.py) in order to grab those relevant image and audio files. Dogs’ facial expressions and barking analysis are performed at this stage, and the predicted results will be returned to Node.js. The RESTful API stores the predicted result in the MySQL database and further obtains a recommended action from the database records according to the respective input.

For instance, if the predicted result is “sick” for the dog’s condition, the MySQL database should return the owner’s email; additionally, an alert message will be delivered to the owner. On the other hand, if the predicted result shows “boring”, the interface of the Android device will be switched on and connect to one of the dog’s friends in its network. When an active account (dogs that are near their respective devices through sensing) is chosen, dogs are able to meet each other, and the barking records from both sides will be shared when they are captured. Furthermore, Google data studio is used to compile and visualize dogs’ conditions. Dog owners can even access an interactive dashboard and monitor their pets remotely through the system.

## 4. Affective Recommender Framework

**Figure 2:**
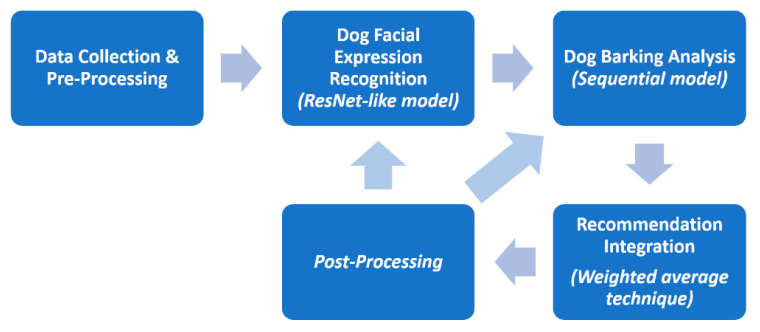
The overall framework of the proposed affective recommender.

The proposed affective recommender engine aims to provide an alert or early notification services to inexperienced dog owners through the dog’s facial expression and barking analysis. There are several alternatives or auxiliary elements for assessing dogs’ expression and behavior, such as ear and tail positions, mouth conditions, and body postures [47]. However, the facial expression of animals is still the richest channel that is used for expressing emotions [48]. Recognizing these visual signal expressions as emotional communication is important because emotions describe the internal state that is influenced by the central nervous system in response to an event [49]. Most experienced dog owners can equally identify the explicit dog’s facial expression; thus, these human experts help in verifying the recognition performances easily later [50]. In addition to facial expressions, acoustic parameters such as dog barks showed promising performance in recognition tasks. Dog barking analysis can achieve more than human-level performance when classifying the context of a dog’s bark [51]. The motivation for the proposed affective recommender engine is to combine both dog facial expressions and barking analysis for better dog emotion recognition. The recommender engine consists of the following modules, as shown in Figure 2.

### 4.1. Data Collection and Pre-Processing

**Figure 3:**
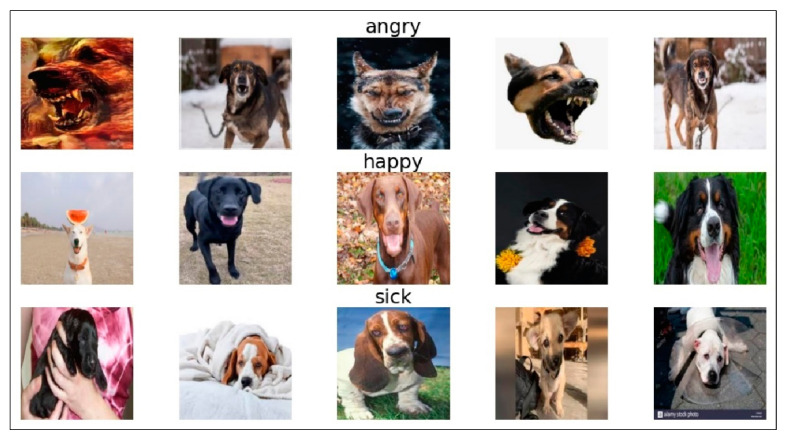
Sample images of dogs with various expressions.

Before training, images of dogs with various expressions were collected and divided into three categories: happy, angry, and sick. The collection of the images was performed according to the description in [49] as shown in Table 1. A Python script with an automated bot was written to download images of dogs from Google Images and save them in local storage. Images that were not related to the categories were removed, and the images were resized to a specific resolution of 224 × 224, as shown in Figure 3. To start building the recognition model, images were split into training, validation, and test data. Since the dataset was small, data augmentation was performed to replace the original batch of images with a randomly transformed batch.

### 4.2. Dogs’ Facial Expression Recognition

**Figure 4:**
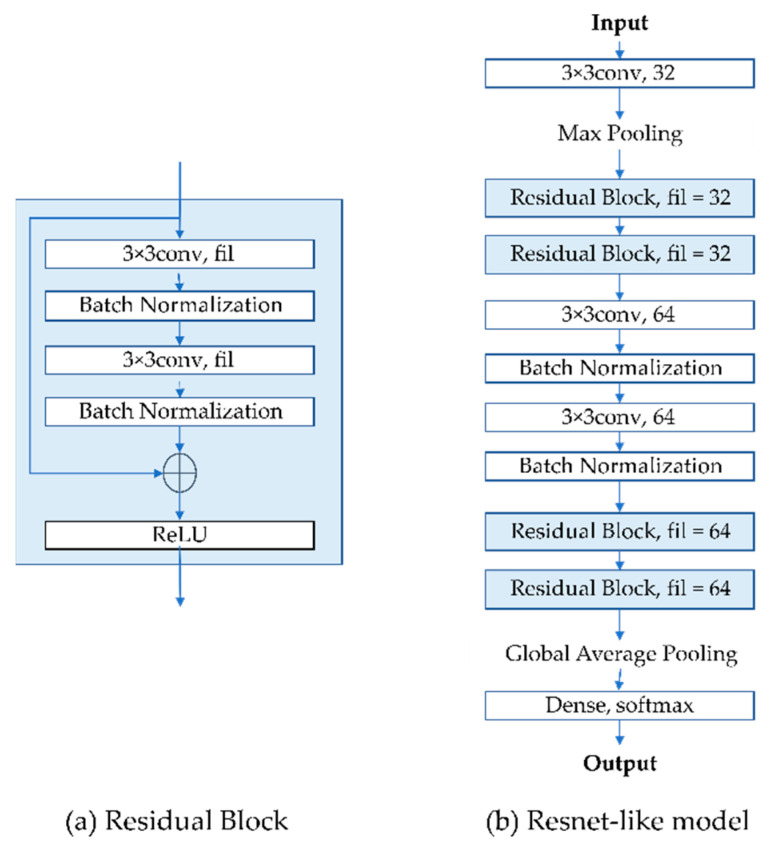
The ResNet-like model consists of twelve convolutional layers. (**a**) Residual block of the ResNet-like model with two convolutional and batch normalization layers, and (**b**) the whole structure of the ResNet-like model.

The idea of the deep learning algorithm Residual Neural Network (ResNet) [23] was adopted to train the image recognition engine due to its robust performance in image recognition. As described in the paper [23], the residual learning was integrated into every few stacked layers, which is known as the building block shown in the equation below:

(1) $$y=F\left(x,\;\left\{W_{i}\right\}\right)+x\;$$

where x and y are the input and output vectors of the layers considered, and $F\left(x,\;\left\{W_{i}\right\}\right)$ is the multiple convolutional layers in the residual block of the ResNet. To demonstrate the feasibility of the proposed framework, a ResNet-like model which consists of twelve layers (as shown in Figure 4b) was implemented. The ResNet-like model consists of four residual blocks, each of which consists of two convolutional layers and batch normalization, as shown in Figure 4a. In each convolutional layer, the filters are 32 and 64, respectively. There are two convolutional layers included after the two residual blocks of the filter size 32. To construct the model, the Adam optimizer [52] that performs fast optimization efficiently was chosen. In addition, the sparse categorical cross entropy was selected as the loss function where a single integer was labeled for each category rather than a whole vector. The expression “happy” is labeled as 0, “angry” is labeled as 1, and “sick” is labeled as 2. Global average pooling and a dense layer were implemented at the end of the model.

**Figure 5:**
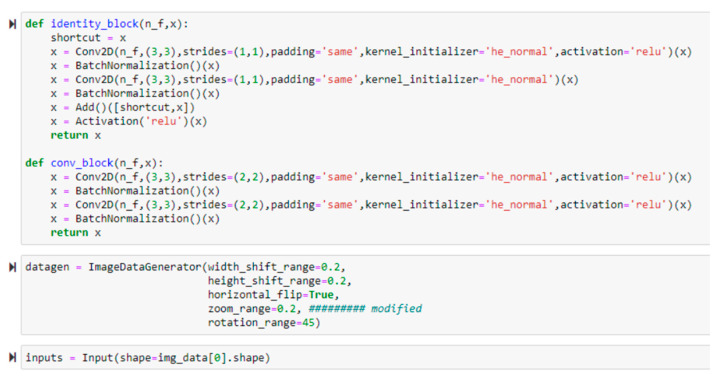
Building the residual block of the ResNet-like model.

As shown in Figure 5, the code in the first block shows the function that generates a ResNet-like network. The second block indicates a function of the ImageDataGenerator that performs the data augmentation over the original batch images. The output from the data augmentation is selected during the training stage with the convolutional neural network (CNN) model. To determine the hyperparameters of the ResNet-like model, successive experiments were conducted. The details of the experiments will be discussed in Section 5. From the trained model, the emotions of dogs in input images are able to be identified based on the predicted values.

### 4.3. Dog Barking Analysis

**Figure 6:**
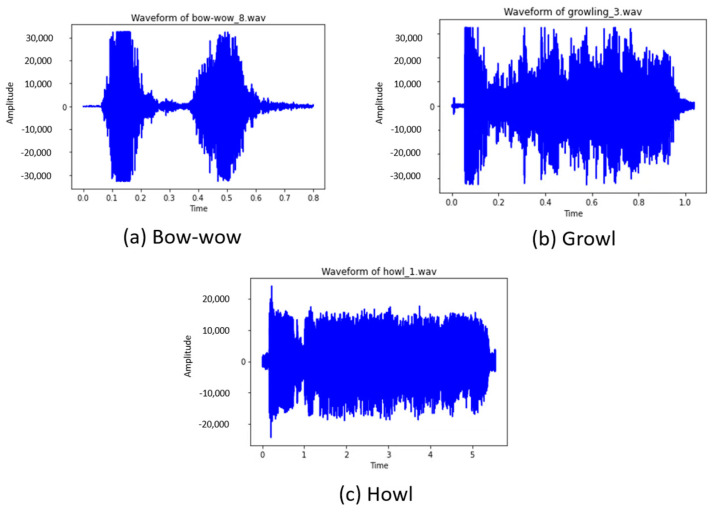
Audio Spectrums of dog barks. (**a**) Dog barks with a bow-wow sound, (**b**) dog barks with a growl sound, and (**c**) dog barks with a howl sound.

After performing dogs’ facial expression recognition, a deep learning-based Sequential model was proposed to analyze dog barks. This study focuses on three types of dog barks: “bow-wow,” “growling,” and “howling.” Each bark corresponds to an expression in the previous dog expression recognition, in which “bow wow” is happy, “growling” is angry, and “howling” is sick. A Python script was also written to download all the required dog barking video files from Google AudioSet and convert them to audio file format (WAV). Later, a software called Audacity was used to study the audio spectrum containing the desired barks, in which the patterns were identified and labeled, as shown in Figure 6. For “bow-wow” class labels, there were two audio spectrums with a gap between the barks. For “growling,” the audio spectrum bounced up and down due to the vibrating sound that a dog makes. For the “howling” class label, the audio spectrum remained constant when the dog howled.

According to the identified patterns, the training dataset was prepared in the preprocessing stage: (1) audio features were extracted from audio files in all directories, and (2) class labels were inserted for each relevant dataset. Once the dataset was completed, a sequential model with four layers was constructed to classify the dog barks. The best epochs for the classification model will be discussed in Section 5. From the trained model, the expressions of the dog from the audio can be identified based on the predicted values.

### 4.4. Recommendation Integration and Post-Processing

**Figure 7:**
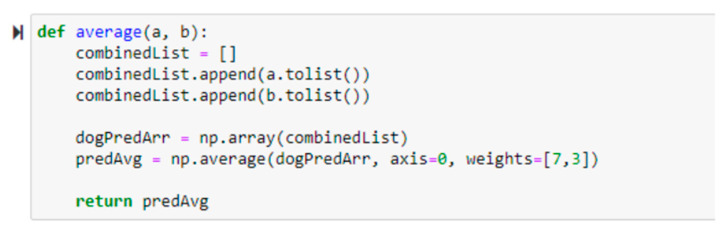
Functions are used to perform weighted average calculations.

**Figure 8:**
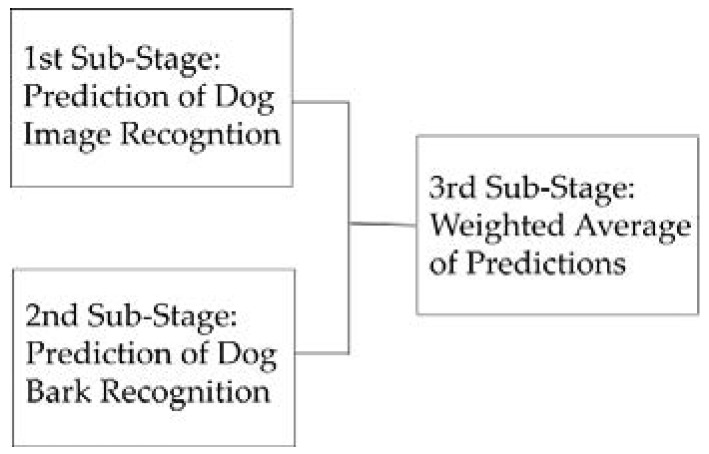
Three sub-stages to predict dogs’ behavior.

A hybrid solution that integrated dogs’ facial expressions and barking analysis was presented earlier. Subsequently, a weighted average technique was adopted to combine the outputs from two predictions. A weighted average function as shown in Figure 7 was chosen. In general, the proposed recommender system involves three sub-stages in predicting dogs’ behavior: the first sub-stage performs dog image recognition; the second sub-stage operates dog bark recognition; the third sub-stage integrates both recognition outputs with a weighted average technique, as shown in Figure 8.

The prediction outputs from the two trained models were combined to improve the result. Each input produces its predicted value for each category (“bow-wow,” “growling,” and “howling”) from both models. A weighted average was implemented to calculate the weight of the predictions. The calculation is shown in the equation below:

(2) $$Average\;weight=\frac{\left(7*x\right)+\left(3*y\right)}{10}\;$$

where x is the predicted value for a specific category of dogs’ facial expression recognition, y is the predicted value for a specific category of dog barking recognition, and x is corresponding to y. The dogs’ facial expression recognition model is weighted higher than the dog barking recognition model because it has higher accuracy. By comparing the average weights of the categories, the one with the highest values will be the predicted dog emotion or behavior.

As illustrated in Figure 2, the prediction outputs from the recommendation integration will provide feedback to respective recognition models in post-processing. The feedback includes user satisfaction and respective confidence values for further recommendation engine improvement and fine-tuning. The performance of the proposed affective recommender framework will be shown in Section 5.

### 4.5. Building Dogs’ Social Network

**Figure 9:**
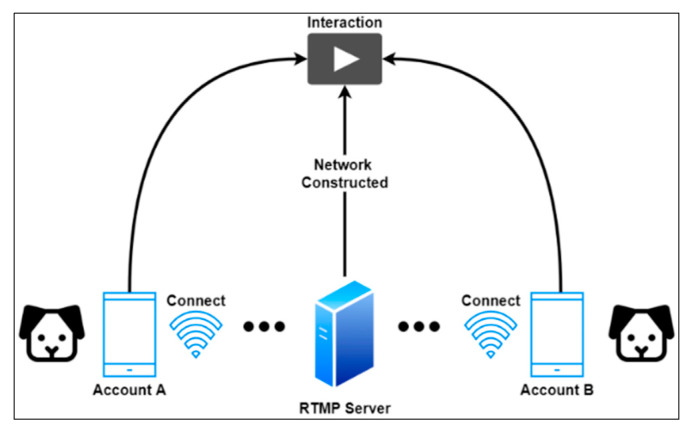
The distributed system architecture of proposed dogs’ social network.

**Figure 10:**
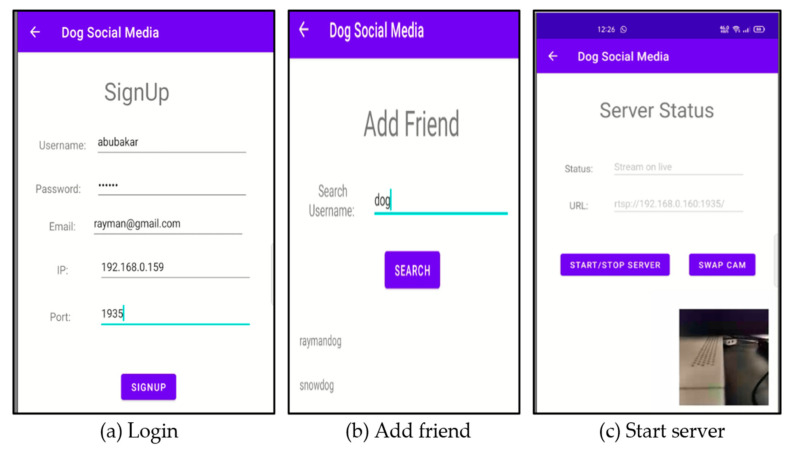
User interface of the developed dogs’ social network application: (**a**) login page of the application to set up live streaming, (**b**) add friend into the database, and (**c**) start the RTMP server.

As mentioned earlier, a social network for dogs is proposed to relieve separation anxiety, especially for those dogs that are left alone. A distributed system architecture is proposed to enable dogs to communicate with each other remotely, as shown in Figure 9. As described in Section 3, the developed mobile app in this study not only predicts dogs’ behavior but also connects with other users’ remote RTMP servers for interaction. Rather than installing complicated equipment, the proposed application allowed any household with dogs to create a smart home environment for their pets by setting up a mobile phone. Owners create a dog account in the application by providing the required information such as username, password, email, RTMP IP, and port, as shown in Figure 10a. When the account is completed, dogs can have their own friends, just like humans, and their owners can add them to the friend list, as shown in Figure 10b.

**Figure 11:**
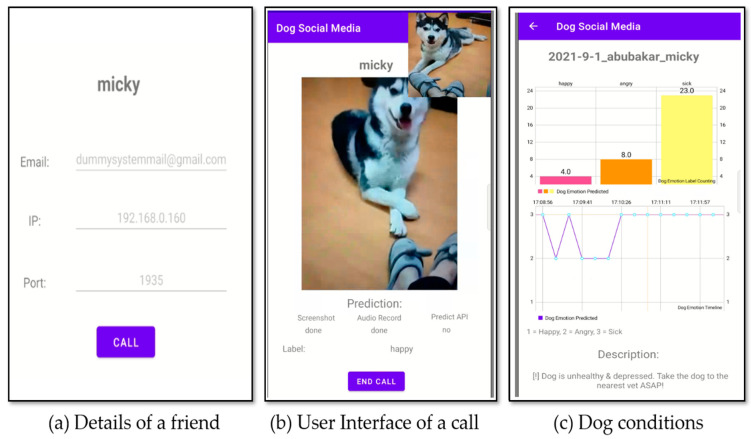
User interface to make a friend call and show the dog’s conditions: (**a**) contact a friend in the name list, (**b**) connect to a dog friend and live streaming, and (**c**) analysis report of the dog emotion.

In order to make a call, there are two important actions: (1) the RTMP server for streaming needs to be activated, as shown in Figure 10c and, (2) the system must check whether the selected friend’s RTMP service is available as well. If it is available, the connection starts to be established and the system prepares the video and audio for live streaming on both sides. This is an automated process if the system detects the dog is “boring” and needs a friend. Figure 11a shows the user interface of the developed mobile app allowing a manual call. It enables the dog owner to manually make a call, just in case there is a need. If the connection to the friend’s RTMP server is successful, the real-time video will be displayed and the audio function will be turned on, as shown in Figure 11b. In the platform setting, the mobile app captures the video and audio from the other side and uploads those data to the cloud for the dog’s behavior training. As shown in Figure 11c, a dog with an unhealthy condition is detected; thus, an alert and notification email are sent to the owner to warn him about the dog’s emotional condition.

## 5. Testing and Discussion

Various experiments have been carried out to train the deep learning models, as mentioned in Section 4, for the proposed affective recommendation engine.

### 5.1. Dog’s Emotion Recognition

**Figure 12:**
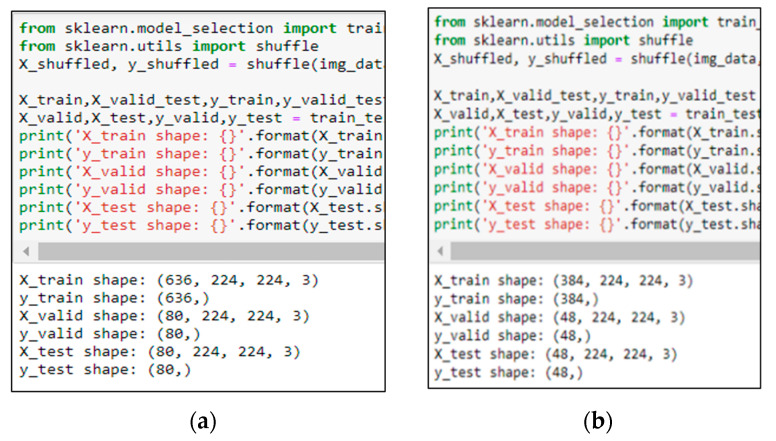
Different numbers of images for training, validation and testing in ResNet-like. (**a**) Dataset with more images and, (**b**) Dataset with fewer images.

The ResNet-like was implemented to recognize dogs’ facial expressions (as described in Section 4.2), and various tests were performed to determine its hyperparameters. Initially, hyperparameters of 200 epochs, batch size of 16, and 0.0005 learning rate were set for training with various dog images as described in Section 4.2. As shown in Figure 12, two sets of images were involved: (1) the dataset of images with a size of 636 for training, 80 for validation and 80 for testing. (2) The dataset of images with a size of 384 for training, 48 for validation and 48 for testing. Based on training prediction results (as shown in Table 2), the accuracies of using fewer images for validation and testing were 70.83% and 66.67%, whereas the accuracies of using more data for validation and testing were 73.75% and 72.50%. The testing was performed using the testing dataset and the accuracy rate of the dataset with fewer images reached 33.33%, which is much lower than the training prediction result, which indicates that overfitting has occurred. The result improved to 53.75% when the dataset with more images was tested. This shows that building the model using the dataset with more images has improved the recognition performance.

**Figure 13:**
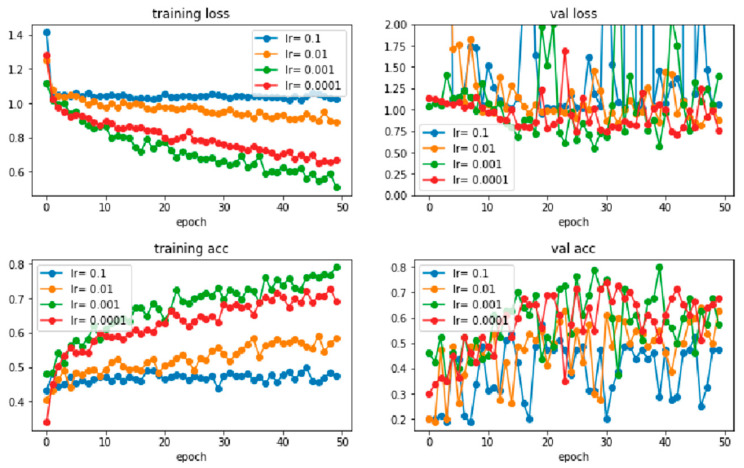
The training loss, validation loss, training accuracy, and validation accuracy of learning rates range from 0.0001 to 0.1 with 50 epochs and a batch size of 16.

**Figure 14:**
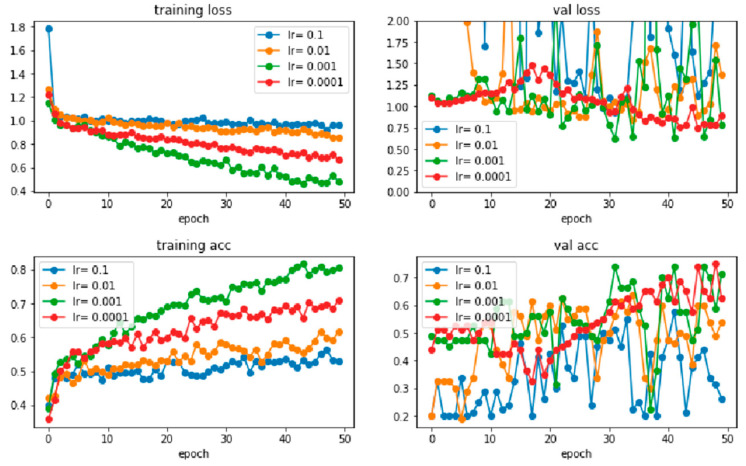
The training loss, validation loss, training accuracy, and validation accuracy of learning rates range from 0.0001 to 0.1 with 50 epochs and a batch size of 32.

Next, the test is continued by tuning the hyperparameters using the dataset with more images, as shown in Table 3. From the table, different learning rates with different numbers of epochs in two common batch sizes (16 and 32) were examined. First, learning rates ranging from 0.0001 to 0.1 with 50 epochs were tested with the batch sizes to determine the appropriate rate. During the training prediction, training loss, validation loss, training accuracy, and validation accuracy were obtained for both batch sizes. Graphs are also plotted, as shown in Figure 13 and Figure 14. In the figures, the loss and accuracy for learning rates of 0.01 and 0.1 are not ideal when compared to the learning rates of 0.001 and 0.0001, where the loss is higher, and the accuracy is lower. When comparing the performance of all learning rates, the learning rate of 0.0001 shows continuous and steady improvement for both batch sizes. For example, in the validation loss, learning rates of 0.001, 0.01, and 0.1 fluctuate more than the learning rate of 0.0001, as shown in Figure 13 and Figure 14. In other words, the learning rate of around 0.0001 is appropriate for the training of this model, where learning rates of between 0.0001 and 0.0005 are set for both batch sizes with the observation by increasing the number of epochs gradually for the next tuning step.

**Figure 15:**
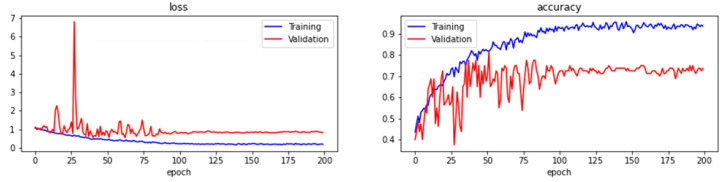
The training loss, validation loss, training accuracy, and validation accuracy of learning rates range from 0.0005 with maximum epochs of 200 and batch size of 16.

**Figure 16:**
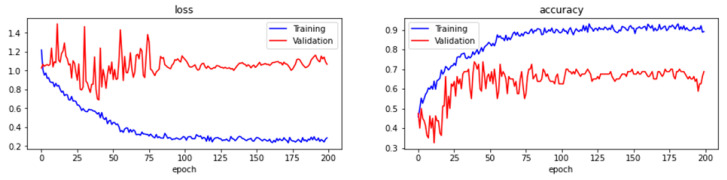
The training loss, validation loss, training accuracy, and validation accuracy of learning rates range from 0.0005 with maximum epochs of 200 and batch size of 32.

Figure 15 and Figure 16 are the training prediction results with batch sizes of 16 and 32. From the figures, the loss and accuracy fluctuate and become consistent starting around 75 epochs. The training and validation losses are consistent when the number of epochs with the batch size of 16 increases (as shown in Figure 15), whereas the training and validation loss function values deviate from each other with the batch size of 32 as shown in Figure 16. Later, the testing was conducted using the test dataset, and the accuracy and loss of batch size 16 reached 53.75% and 0.6038 while the accuracy and loss of batch size 32 reached 43.75% and 0.6629. As shown in Table 4, the result reveals that the model trained with batch size 16 is better than the batch size of 32 as it achieves better accuracy and lower loss. In summary, a learning rate of between 0.0001 and 0.0005, 200 epochs, and a batch size of 16 are the hyperparameters discovered to build the ResNet-like model in this system. The model is compared to VGG16 [53] as well when using the same settings of hyperparameters to evaluate the performance. The tests were also carried out in batch 16 and batch 32 for VGG16 and compared with ResNet-like in Table 4. As noticed in the table, the overall performance of ResNet-like is better than VGG16 since all the accuracies for VGG16 are less than 50% and the loss values are larger than 1.

**Figure 17:**
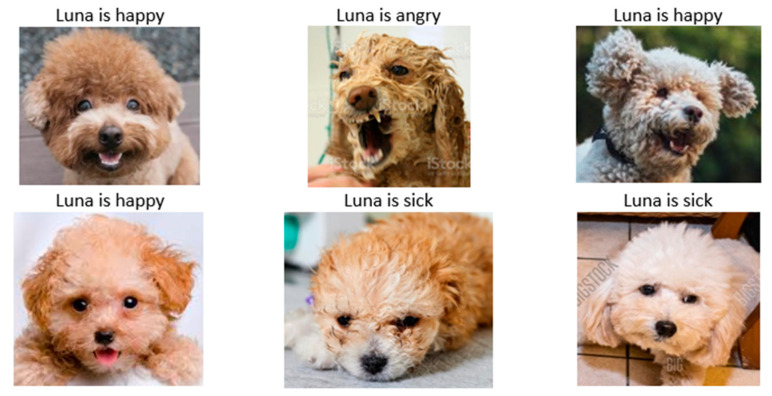
Prediction results of the emotions of a dog named Luna through the constructed ResNet-like model. Luna’s emotion is predicted correctly in all images.

With the constructed model, the test proceeded on the sample of dog images to predict dog emotions, as shown in Figure 17. The images of a dog named Luna were collected and tested on the model. Luna’s emotions were predicted correctly in all images.

### 5.2. Dog Barking Emotion Recognition and Weighted Average for Dogs’ Behavior Prediction

**Figure 18:**
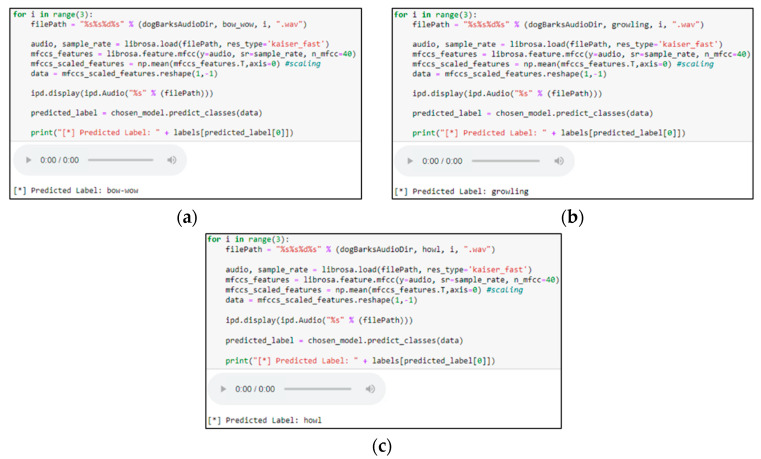
Prediction results of three types of dog barks on the test dataset using a trained model. (**a**) Prediction of bow-wow, (**b**) Prediction of growling and, (**c**) Prediction of Howl.

A sequential model was implemented to recognize dog barks (as described in Section 4.3) and simple tests were performed to determine its hyperparameters. Initially, 100 epochs and a batch size of 32 were set for training, and the validation of the training became consistent after starting a second epoch based on observation. Then, the model was tested with the test dataset, and the classification accuracy showed 75%. As shown in Figure 18, the model is able to predict the types of dog barks based on the provided audio test files.

**Figure 19:**
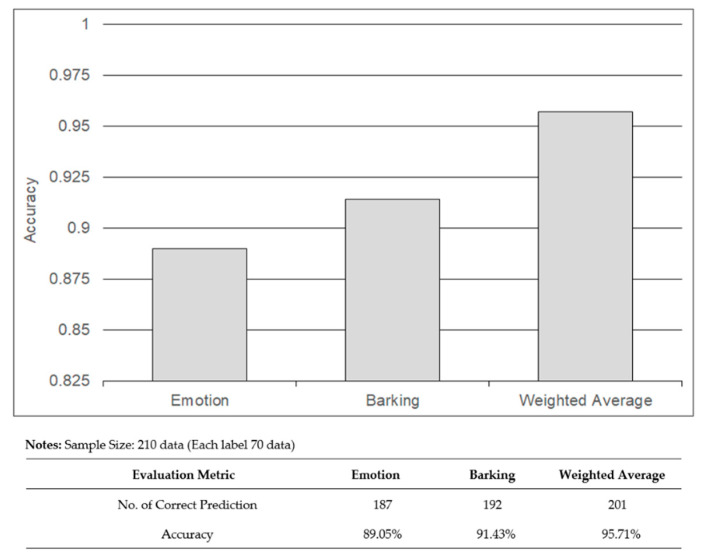
Prediction accuracy obtained from dogs′ emotions, dog barking, and weighted average. The weighted average which combines the prediction value of dogs’ emotion and dog barking shows the highest accuracy when compared to emotion and barking.

**Figure 20:**
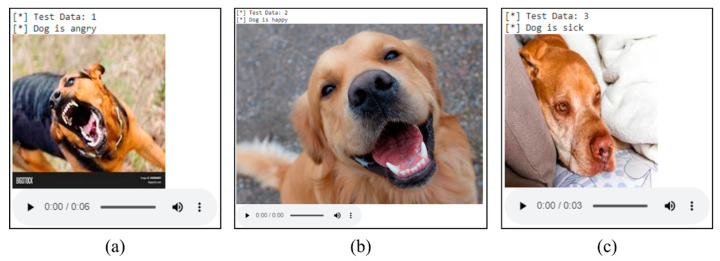
Samples of test data (angry, happy, and sick) using the weighted average technique. The outputs shows (**a**) Dog is angry, (**b**) Dog is happy and, (**c**) Dog is sick.

As explained in Section 4, the predicted outputs of the two trained models were combined through a weighted average using Equation (2) to enhance the prediction of dogs’ behavior. The predicted output showed the dogs’ behaviors which have been categorized as happy, angry, and sick. A total of 70 sample data files for each class label were prepared for testing, which are 210 dog images and 210 dog barking audio files in total. Figure 19 shows the accuracy of the predicted output for dog emotions, dog barking, and weighted average. The weighted average had the highest accuracy with 201 samples (95.70%) correctly predicting the dogs’ behavior, while 187 images (89%) and 192 barks (91.40%) correctly predicted the dogs’ behavior. Figure 20 shows three samples of the test data that correctly predict the dogs’ behavior through the weighted average. In summary, the combination of dog emotions and dog barking improves the prediction accuracy of dogs’ behavior in the three categories of happy, angry, and sick.

## 6. Conclusions

Dogs are good companions for humans; they have a close relationship with their owners. However, dogs may face separation anxiety when they are apart from their owners for a long period of time and even develop disruptive behavior. Therefore, a novel cloud-based smart environment dog social network is proposed to solve this problem for dogs that live around the household. A mobile app for smartphones was developed to predict the dogs’ behavior, and smartphones are used as communication devices to connect with different dog friends from different households. The ResNet-like model is used for dog emotion recognition in predicting dogs’ behavior. A series of experiments were carried out to determine the hyperparameters of the ResNet-like model which found a learning rate of between 0.0001 and 0.0005, 200 epochs, and a batch size of 16. The proposed model was able to achieve 53.75% accuracy a 60.38% loss. The sequential model is used for dog barking recognition to predict the dog’s behavior as well. The model was tested with the test dataset and the classification accuracy was shown to be 75%. Later, the weighted average technique (a combination of the prediction values of dog emotion recognition and dog barking recognition) was chosen to improve the prediction output, and it achieved an accuracy of 95.70%. On the other hand, the RTMP server is implemented as a platform to connect dog friends in a list using smartphones. Once RTMP is established, dogs can interact with each other, and it will trigger notification messages to owners once a sick dog is detected. In future work, dog pose recognition could be included to further improve the classification accuracy of the proposed affective recommender system. Due to the limitations of current data acquisition, multimodal training datasets should be applied for subsequent experiments to improve the recognition output. Furthermore, we may concentrate on the validity of the proposed system for various types of dogs and environments. The feasibility of the proposed solution could be one of the research directions.

## Figures and Tables

**Table 1:** Dog facial expressions (happy, angry, and sick) characteristics [49].

Facial Expression	Eyes	Ears	Mouth/Teeth
Happy	Wide open, merry looking, raised eyebrows	Perked-up and forward, or relaxed	Mouth relaxed and slightly open, teeth covered, excited panting, possible lip-licking
Angry	Narrow or staring challengingly	Forward or back, close to head	Lips open, drawn back to expose teeth bared in a snarl, possible jaw snapping
Sick	Eyelids semi-closed with tearing, raised eyebrows, simulating large eyes, sad gaze	Distance between ears tends to widen	Contracted, giving the appearance of wrinkles on the face

**Table 2:** The comparison of performance between smaller and larger sample sizes of data using ResNet-like batch 16. Larger sample size of data has better performance (as highlighted) compared to less data sample size.

	Evaluation Metric	Less Data Sample Size	More Data Sample Size
Training	Accuracy	70.83%	**73.75%**
	Loss	0.8192	**0.8289**
Validation	Accuracy	66.67%	**72.50%**
	Loss	0.8482	**0.6038**
Test	Accuracy	33.33%	**53.75%**
	Loss	0.8482	**0.6038**

**Table 3:** Testing of the ResNet-like model with different hyperparameters using dataset with more images. The numbers in bold number are the hyperparameters discovered to build the ResNet-like model in this system.

Learning Rate	Batch Size of 16		Batch Size of 32	
0.1	50 epochs	**200 epochs**	50 epochs	200 epochs
0.01				
0.001				
**0.0001**				

**Table 4:** The comparison of performances between hyperparameter batches 16 and 32. ResNet-like trained with batch size 16 is better than the batch size of 32 and it is also better than VGG16 as highlighted.

Hyper-Parameter		Evaluation Metric	ResNet-like	VGG16
	Training	Accuracy	**73.75%**	47.50%
		Loss	**0.8289**	1.0408
**Batch 16**	Validation	Accuracy	**72.50%**	47.50%
		Loss	**0.6038**	1.0408
	Test	Accuracy	**53.75%**	35.27%
		Loss	**0.6038**	1.0408
	Training	Accuracy	**68.75%**	47.50%
		Loss	**1.0672**	1.0408
**Batch 32**	Validation	Accuracy	**72.50%**	47.50%
		Loss	**0.6629**	1.0408
	Test	Accuracy	**43.75%**	38.16%
		Loss	**0.6629**	1.0408

## Data Availability

There are no data applicable in this study.

## References

[1] Rasch K. (2014). An Unsupervised Recommender System for Smart Homes. J. Ambient Intell. Smart Environ..

[2] Ojagh S., Malek M.R., Saeedi S., Liang S. (2020). A Location-Based Orientation-Aware Recommender System Using IoT Smart Devices and Social Networks. Future Gener. Comput. Syst..

[3] Mishra P., Gudla S.K., ShanBhag A.D., Bose J. (2020). Alternate Action Recommender System Using Recurrent Patterns of Smart Home Users. Proceedings of the 2020 IEEE 17th Annual Consumer Communications & Networking Conference (CCNC).

[4] Gladence L.M., Anu V.M., Rathna R., Brumancia E. (2020). Recommender System for Home Automation Using IoT and Artificial Intelligence. J. Ambient Intell. Humaniz. Comput..

[5] Altulyan M., Yao L., Wang X., Huang C., Kanhere S.S., Sheng Q.Z. (2021). A Survey on Recommender Systems for Internet of Things: Techniques, Applications and Future Directions. Comput. J..

[6] Liu H., Zheng C., Li D., Shen X., Lin K., Wang J., Zhang Z., Zhang Z., Xiong N.N. (2021). EDMF: Efficient Deep Matrix Factorization with Review Feature Learning for Industrial Recommender System. IEEE Trans. Ind. Inform..

[7] Liu H., Zheng C., Li D., Zhang Z., Lin K., Shen X., Xiong N.N., Wang J. (2022). Multi-Perspective Social Recommendation Method with Graph Representation Learning. Neurocomputing.

[8] Li D., Liu H., Zhang Z., Lin K., Fang S., Li Z., Xiong N.N. (2021). CARM: Confidence-Aware Recommender Model via Review Representation Learning and Historical Rating Behavior in the Online Platforms. Neurocomputing.

[9] Rodríguez Fernández M., Cortés García A., González Alonso I., Zalama Casanova E. (2016). Using the Big Data Generated by the Smart Home to Improve Energy Efficiency Management. Energy Effic..

[10] Hossain M.S., Rahman M.A., Muhammad G. (2017). Cyber–Physical Cloud-Oriented Multi-Sensory Smart Home Framework for Elderly People: An Energy Efficiency Perspective. J. Parallel Distrib. Comput..

[11] Lye G.X., Cheng W.K., Tan T.B., Hung C.W., Chen Y.-L. (2020). Creating Personalized Recommendations in a Smart Community by Performing User Trajectory Analysis through Social Internet of Things Deployment. Sensors.

[12] Wang R., Liu Y., Zhang P., Li X., Kang X. (2020). Edge and Cloud Collaborative Entity Recommendation Method towards the IoT Search. Sensors.

[13] Cheng W.K., Ileladewa A.A., Tan T.B. (2019). A Personalized Recommendation Framework for Social Internet of Things (SIoT). Proceedings of the 2019 International Conference on Green and Human Information Technology (ICGHIT).

[14] Gardner B. (2015). A Review and Analysis of the Use of ‘Habit’in Understanding, Predicting and Influencing Health-Related Behaviour. Health Psychol. Rev..

[15] Alsalemi A., Sardianos C., Bensaali F., Varlamis I., Amira A., Dimitrakopoulos G. (2019). The Role of Micro-Moments: A Survey of Habitual Behavior Change and Recommender Systems for Energy Saving. IEEE Syst. J..

[16] Yang H., Lee W., Lee H. (2018). IoT Smart Home Adoption: The Importance of Proper Level Automation. J. Sens..

[17] McCrave E.A. (1991). Diagnostic Criteria for Separation Anxiety in the Dog. Vet. Clin. N. Am. Small Anim. Pract..

[18] Wang H., Atif O., Tian J., Lee J., Park D., Chung Y. (2022). Multi-Level Hierarchical Complex Behavior Monitoring System for Dog Psychological Separation Anxiety Symptoms. Sensors.

[19] Pet Ownership in Asia. https://insight.rakuten.com/pet-ownership-in-asia/.

[20] How to Manage Anti-Social Behavior in Your Pandemic Dog. https://www.nextavenue.org/separation-anxiety-in-dog/.

[21] 6 Ways To Ease Post-Pandemic Separation Anxiety in Pets|Mars, Incorporated. https://www.mars.com/news-and-stories/articles/6-ways-ease-post-pandemic-separation-anxiety-pets.

[22] Shannon L. (2020). Dog Gone: How to Handle Your Pet’s Post—Covid Separation Anxiety.

[23] He K., Zhang X., Ren S., Sun J. Deep Residual Learning for Image Recognition. Proceedings of the IEEE Conference on Computer Vision and Pattern Recognition.

[24] Rashidi P., Cook D.J., Holder L.B., Schmitter-Edgecombe M. (2010). Discovering Activities to Recognize and Track in a Smart Environment. IEEE Trans. Knowl. Data Eng..

[25] Belghini N., Gouttaya N., Bouab W., Sayouti A. (2016). Pervasive Recommender System for Smart Home Environment. Int. J. Appl. Inf. Syst..

[26] Thakur N., Han C.Y. (2018). A Context-Driven Complex Activity Framework for Smart Home. Proceedings of the 2018 IEEE 9th Annual Information Technology, Electronics and Mobile Communication Conference (IEMCON).

[27] Felfernig A., Polat-Erdeniz S., Uran C., Reiterer S., Atas M., Tran T.N.T., Azzoni P., Kiraly C., Dolui K. (2019). An Overview of Recommender Systems in the Internet of Things. J. Intell. Inf. Syst..

[28] Corujo L.A., Kieson E., Schloesser T., Gloor P.A. (2021). Emotion Recognition in Horses with Convolutional Neural Networks. Future Internet.

[29] Voorend R.W.A. (2021). Deep Unsupervised Representation Learning For Animal Activity Recognition.

[30] Ladha C., Hammerla N., Hughes E., Olivier P., Ploetz T. Dog’s Life: Wearable Activity Recognition for Dogs. Proceedings of the 2013 ACM International Joint Conference on Pervasive and Ubiquitous Computing.

[31] Iwashita Y., Takamine A., Kurazume R., Ryoo M.S. (2014). First-Person Animal Activity Recognition from Egocentric Videos. Proceedings of the 2014 22nd International Conference on Pattern Recognition.

[32] Pons P., Jaen J., Catala A. Developing a Depth-Based Tracking System for Interactive Playful Environments with Animals. Proceedings of the 12th International Conference on Advances in Computer Entertainment Technology.

[33] Kamminga J.W., Bisby H.C., Le D.V., Meratnia N., Havinga P.J. Generic Online Animal Activity Recognition on Collar Tags. Proceedings of the 2017 ACM International Joint Conference on Pervasive and Ubiquitous Computing and Proceedings of the 2017 ACM International Symposium on Wearable Computers.

[34] Casella E., Khamesi A.R., Silvestri S. (2019). Smartwatch Application for Horse Gaits Activity Recognition. Proceedings of the 2019 IEEE International Conference on Smart Computing (SMARTCOMP).

[35] Siniscalchi M., Quaranta A., Rogers L.J. (2008). Hemispheric Specialization in Dogs for Processing Different Acoustic Stimuli. PLoS ONE.

[36] Quaranta A., d’Ingeo S., Amoruso R., Siniscalchi M. (2020). Emotion Recognition in Cats. Animals.

[37] Totakura V., Janmanchi M.K., Rajesh D., Hussan M.T. (2020). Prediction of Animal Vocal Emotions Using Convolutional Neural Network. Int. J. Sci. Technol. Res..

[38] Singh B.K., Dua T., Sharma D.P., Changare A.A. (2021). Animal Emotion Detection and Application. Data Driven Approach towards Disruptive Technologies.

[39] Caeiro C., Guo K., Mills D. (2017). Dogs and Humans Respond to Emotionally Competent Stimuli by Producing Different Facial Actions. Sci. Rep..

[40] Liu T., Yang B., Liu H., Ju J., Tang J., Subramanian S., Zhang Z. (2022). GMDL: Toward Precise Head Pose Estimation via Gaussian Mixed Distribution Learning for Students’ Attention Understanding. Infrared Phys. Technol..

[41] Liu H., Fang S., Zhang Z., Li D., Lin K., Wang J. (2021). MFDNet: Collaborative Poses Perception and Matrix Fisher Distribution for Head Pose Estimation. IEEE Trans. Multimed..

[42] Liu H., Liu T., Zhang Z., Sangaiah A.K., Yang B., Li Y. (2022). ARHPE: Asymmetric Relation-Aware Representation Learning for Head Pose Estimation in Industrial Human–Computer Interaction. IEEE Trans. Ind. Inform..

[43] Zhang S., Yao L., Sun A., Tay Y. (2019). Deep Learning Based Recommender System: A Survey and New Perspectives. ACM Comput. Surv. CSUR.

[44] Hassan M.M., Uddin M.Z., Mohamed A., Almogren A. (2018). A Robust Human Activity Recognition System Using Smartphone Sensors and Deep Learning. Future Gener. Comput. Syst..

[45] Kamminga J.W., Le D.V., Havinga P.J.M. Towards Deep Unsupervised Representation Learning from Accelerometer Time Series for Animal Activity Recognition. Proceedings of the 6th Workshop on Mining and Learning from Time Series.

[46] Bocaj E., Uzunidis D., Kasnesis P., Patrikakis C.Z. (2020). On the Benefits of Deep Convolutional Neural Networks on Animal Activity Recognition. Proceedings of the 2020 International Conference on Smart Systems and Technologies (SST).

[47] Ferres K., Schloesser T., Gloor P.A. (2022). Predicting Dog Emotions Based on Posture Analysis Using DeepLabCut. Future Internet.

[48] Neethirajan S. (2021). Happy Cow or Thinking Pig? Wur Wolf—Facial Coding Platform for Measuring Emotions in Farm Animals. AI.

[49] Mota-Rojas D., Marcet-Rius M., Ogi A., Hernández-Ávalos I., Mariti C., Martínez-Burnes J., Mora-Medina P., Casas A., Domínguez A., Reyes B. (2021). Current Advances in Assessment of Dog’s Emotions, Facial Expressions, and Their Use for Clinical Recognition of Pain. Animals.

[50] Blumrosen G., Hawellek D., Pesaran B. Towards Automated Recognition of Facial Expressions in Animal Models. Proceedings of the IEEE International Conference on Computer Vision Workshops (ICCVW).

[51] Hantke S., Cummins N., Schuller B. (2018). What Is My Dog Trying to Tell Me? The Automatic Recognition of the Context and Perceived Emotion of Dog Barks. Proceedings of the 2018 IEEE International Conference on Acoustics, Speech and Signal Processing (ICASSP).

[52] Kingma D.P., Ba J. (2014). Adam: A Method for Stochastic Optimization. arXiv.

[53] Simonyan K., Zisserman A. (2014). Very Deep Convolutional Networks for Large-Scale Image Recognition. arXiv.

